# Antimicrobial and Cytoprotective Effects of Tea Extracts Against *Escherichia coli*-Producing Colibactin Toxin Infections

**DOI:** 10.3390/antibiotics14090886

**Published:** 2025-09-02

**Authors:** Wipawadee Teppabut, Yingmanee Tragoolpua, Thida Kaewkod

**Affiliations:** 1 Department of Biology, Faculty of Science, Chiang Mai University, Chiang Mai 50200, Thailand; vipawadee_th@cmu.ac.th (W.T.); yingmanee.t@cmu.ac.th (Y.T.); 2 Master of Science Program in Applied Microbiology (International Program), Faculty of Science, Chiang Mai University, Chiang Mai 50200, Thailand; 3 Natural Extracts and Innovative Products for Alternative Healthcare Research Group, Faculty of Science, Chiang Mai University, Chiang Mai 50200, Thailand

**Keywords:** antibacterial, colibactin toxin, cytoprotective effects, *Escherichia coli*, tea

## Abstract

**Background/Objectives**: *Camellia sinensis* (L.) Kuntze or tea contains bioactive compounds such as catechin and caffeine, known for their antimicrobial and health-promoting properties. Colibactin-producing *Escherichia coli* are linked to genotoxicity in colon epithelial cells, potentially contributing to colorectal disease. This study aimed to evaluate the inhibitory effects of tea extracts (green, oolong, and black) and the phytochemicals catechin and caffeine on *E. coli* pathogenesis mediated by colibactin toxins, including transient infections, DNA damage, and cell cycle alterations in Caco-2 colon cells. **Methods**: Tea extracts were analyzed by HPLC for phytochemical content. Their antimicrobial activity against colibactin-producing *E. coli* (ATCC 25922) was assessed. Caco-2 cells were infected with the bacteria and treated with tea extracts or compounds. Cell viability was measured by MTT assay, DNA damage was measured by alkaline comet assay, and the expression of *CDK-1*, *CDK-2*, and *Ki-67* genes was measurd by qRT-PCR. **Results**: Tea extracts and catechin inhibited colibactin-producing *E. coli* and significantly protected Caco-2 cells. Oolong tea showed the highest protection (90.78 ± 2.76%), with others maintaining viability above 80%. DNA damage was markedly reduced, and cell cycle regulation improved. All extracts upregulated *CDK-1* and downregulated *CDK-2*, aiding in cell cycle restoration. *Ki-67* expression indicated enhanced cell proliferation during infection. **Conclusions**: This study highlights new findings showing that tea extracts, including green, oolong, and black tea, as well as the tea compounds catechin and caffeine, can protect against DNA damage and help maintain the normal cell cycle of colon cells infected with *E. coli*-producing colibactin toxin. These results support their potential role in preventing and mitigating infections caused by such *E. coli* strains while promoting colon cell health.

## 1. Introduction

*Camellia sinensis* (L.) Kuntze (tea; family *Theaceae*) is the second most widely consumed beverage in the world, following water. It is more popular than coffee, beer, wine, and soft drinks [1,2]. With a history spanning over 2000 years, tea has become an integral part of social culture. Currently, around 70% of the global population consumes approximately 18–20 billion cups of tea each day. To meet the high demand, approximately 2.9 billion tons of tea are produced annually [3]. Tea is made from the buds, young leaves, and tender stalks of the tea plant and is classified into different types based on the degree of fermentation during production: green tea (unfermented), oolong tea (semi-fermented), and black tea (fully fermented) [4]. All tea types originate from the same plant (*C. sinensis*), with heat treatment playing a key role in enzyme inactivation, particularly in the production of green and oolong tea. During oolong tea fermentation, catechin oxidation levels range from 20% to 80%, while black tea features fully oxidized catechins [5,6]. The chemical makeup of tea varies by type, with flavonoids making up 20–30% of its composition. Green tea contains catechins (flavan-3-ols) such as epigallocatechin-3-gallate (EGCG), epigallocatechin (EGC), epicatechin (EC), and epicatechin-3-gallate (ECG). Black tea, on the other hand, is rich in polymerized catechins known as theaflavins, such as theaflavin-3′-gallate, as well as other theaflavins and thearubigins [7]. Additional major theaflavins found in black tea include theaflavin, theaflavin-3-gallate, and theaflavin-3,3′-gallate [8]. Numerous studies have demonstrated the beneficial effects of different types of tea. Tea produced from *C. sinensis* has shown activities such as antidiabetic [9], anticancer [10], antibacterial [11], neuroprotective [12], cardioprotective [13], anti-obesity [14], hepatoprotective [15], and antiviral [16] effects. In this study, we focused on the use of tea extracts to inhibit bacterial infection mechanisms.

Pathogenic enteric bacteria often cause diarrhea and associated illnesses, typically leading to secretory or watery diarrhea following an infection [17]. *Escherichia coli* is the most common bacterial resident of the human gut, and its strains are divided into four primary phylogenetic groups (A, B1, B2, and D), each linked to specific ecological niches [18]. Extraintestinal *E. coli* (ExPEC), classified under group B2, are found in the intestinal microbiota of both humans and animals. These emerging pathogens are known to cause extraintestinal infections, such as urinary tract infections, neonatal meningitis, sepsis, pneumonia, surgical site infections, and infections at other sites outside the intestine [19,20]. Moreover, antimicrobial resistance (AMR) is a significant concern worldwide, particularly concerning *E. coli* strains. A study published in 2024 reported that 58% of *E. coli* isolates exhibited resistance to gentamicin, a commonly used antibiotic [21]. ExPEC strains tend to remain in the colon for longer periods compared to other *E. coli* groups and account for 30–50% of the strains found in the feces of healthy individuals in high-income countries [22,23]. The genomes of ExPEC strains contain the polyketide synthase (*pks*) pathogenicity island, known as the *pks* island, which encodes the 54-kbp colibactin gene cluster responsible for the production of the colibactin toxin [24]. This toxin induces cell cycle arrest at the G2 or M phase, chromosomal aberrations, and double-strand DNA breaks [25]. Cyclin-dependent kinases 1 and 2 (CDK-1 and CDK-2) are essential for controlling cell cycle progression, including transitions from G1 to S, through S, and from G2 to M phases. The activation of CDK-1 and CDK-2 facilitates cell growth and division [26]. Colibactin-induced DNA damage disrupts the cell cycle by causing arrest at the G1/S and G2/M checkpoints, thereby hindering cell growth and regeneration, which supports bacterial colonization. In response to severe DNA damage, cells may undergo apoptosis or develop a senescent phenotype. In cases where DNA repair is incomplete, surviving cells can experience genomic instability, potentially leading to tumor initiation or accelerated tumor progression [27]. Additionally, colibactin-harboring *E. coli* have been found in higher numbers among colorectal cancer (CRC) patients and have been shown to increase tumor formation in various CRC mouse models [28]. This study evaluated the inhibitory effects of tea extracts, including green tea, oolong tea and black tea, and phytochemical compounds of catechin and caffeine on *E. coli* pathogenesis mediated by colibactin toxins, including transient infections, colibactin-induced DNA damage, and their influence on the cell cycle, as observed in Caco-2 colon cell culture models.

## 2. Results

### 2.1. Phytochemical Compounds in Tea Extracts

The phytochemical contents of tea extracts were determined including catechin, EGCG, caffeine and theaflavin by HPLC assays. The results showed that oolong tea extract contained all chemical compounds with high values at 233.475 ± 13.320, 251.839 ± 8.751, 2.658 ± 0.053 and 2.192 ± 0.297 mg/mL, respectively (Table 1). In addition, green tea extract detected a high caffeine contents as 56.909 ± 0.444 mg/mL, followed by catechin and caffeine. For black tea extract was found high content of EGCG with the value of 269.714 ± 7.860 mg/mL (Table 1).

### 2.2. Antibacterial Activity by Agar Well Diffusion

Green tea, oolong tea, and black tea extracts were evaluated for their ability to inhibit *E. coli* ATCC 25922 (a colibactin-producing strain) and *E. coli* K-12 (a non-colibactin-producing strain) using the agar well diffusion technique. The results showed that oolong tea extract at 500 mg/mL exhibited the greatest inhibition of *E. coli* ATCC 25922, with an inhibition zone of 18.33 ± 0.47 mm. Green tea and black tea followed with inhibition zones of 16.33 ± 4.50 mm and 13.67 ± 1.25 mm, respectively (Figure S1 and Table 2). Therefore, phytochemical compounds, specifically catechin (5 mg/mL) and caffeine (5 mg/mL), demonstrated inhibitory effects against *E. coli* ATCC 25922 (Figure S1 and Table 2). For *E. coli* K-12, the inhibition zones were 15.00 ± 1.73 mm for green tea, 16.00 ± 1.00 mm for oolong tea, and 14.00 ± 1.00 mm for black tea (Figure S1 and Table 2). Additionally, 1 mg/mL gentamycin was used as a positive control for bacterial inhibition, resulting in inhibition zones of 23.76 ± 0.47 mm and 26.33 ± 0.58 mm against *E. coli* ATCC 25922 and *E. coli* K-12, respectively (Figure S1 and Table 2).

### 2.3. Cytotoxicity of Tea Extracts and Phytochemical Compounds on Caco-2 Cells

**Figure 1:**
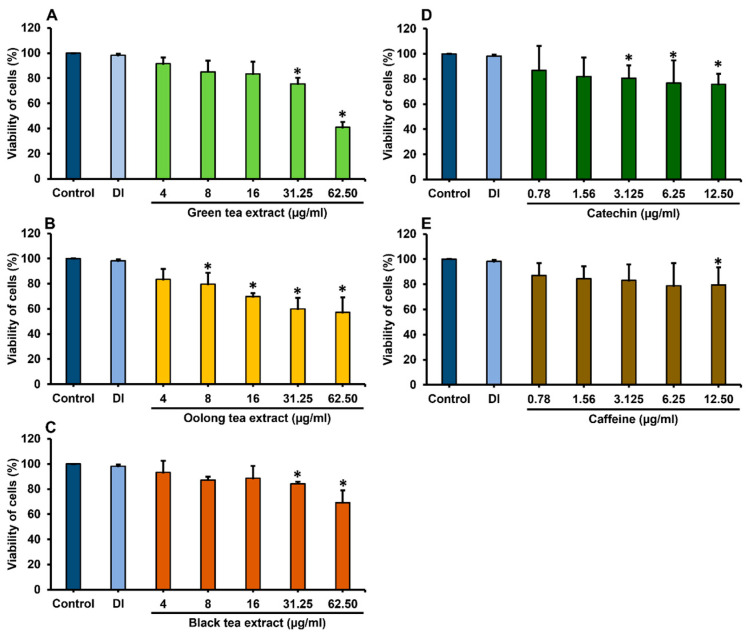
Viability of Caco-2 cells after treatment with green tea (**A**), oolong tea (**B**) and black tea (**C**) extracts and phytochemical compounds of catechin (**D**) and caffeine (**E**) for 48 h. * Data are significantly different compared to control cells (*p* < 0.05). Values are presented as mean ± standard deviation; n = 3 samples. Deionized water (DI) was used as a vehicle control.

The cytotoxicity of tea extracts on Caco-2 cells was determined using the MTT assay to identify non-toxic dose concentrations. After treatment with tea extracts for 48 h, green tea extract at 8 µg/mL and 16 µg/mL did not exhibit toxicity toward Caco-2 cells, with cell viability percentages of 85.09 ± 8.93% and 83.46 ± 9.82%, respectively. Additionally, oolong tea at concentrations of 4 µg/mL and 8 µg/mL, and black tea at concentrations of 16 µg/mL and 32 µg/mL, did not show toxicity to Caco-2 cells, as cell viability remained above 80% (Figure 1). For phytochemical compounds, catechin and caffeine at 0.78–3.13 µg/mL did not have the toxicity to Caco-2 cells with the cell viability above 80% (Figure 1).

### 2.4. Inhibitory Effects of Tea Extracts and Phytochemical Compounds on Transient Infection of Caco-2 Cells by E. coli ATCC 25922

**Figure 2:**
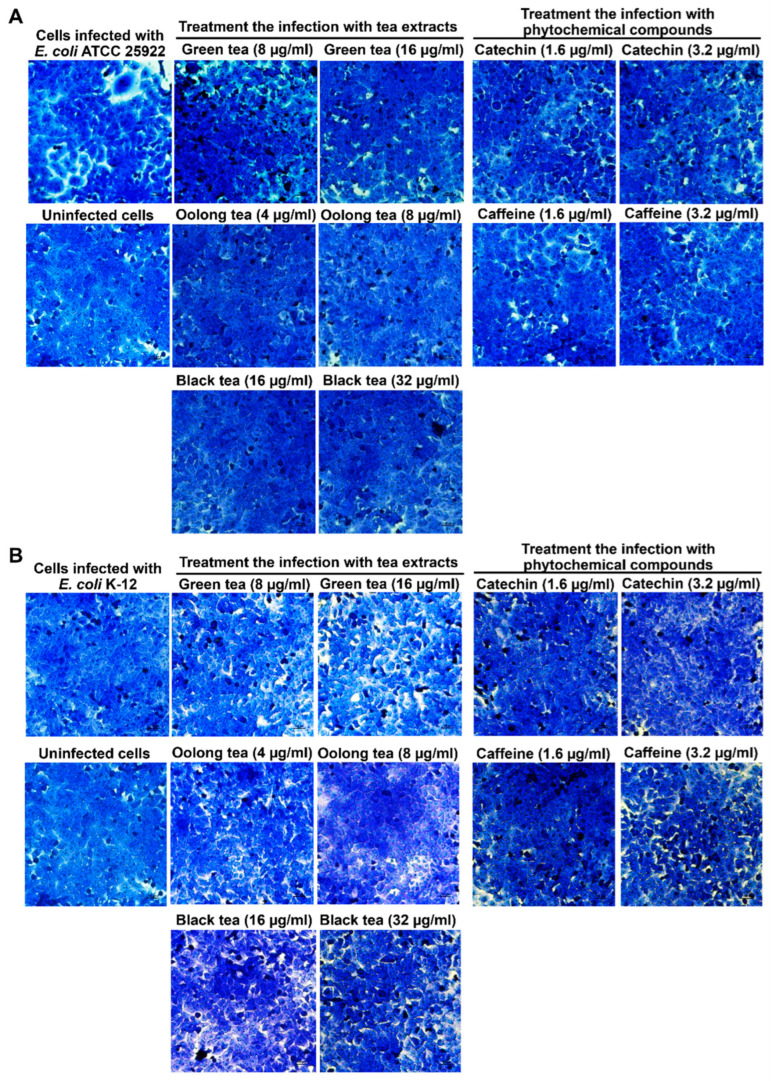
Efficacy of tea extracts and phytochemical compounds for inhibition of transient infection with *E. coli* ATCC 25922 (**A**) at a MOI of 400 on Caco-2 cells for 4 h when compared to control infected epithelial cells and transient infection with *E. coli* K-12 (**B**).

The ability of tea extracts to inhibit transient infection of Caco-2 cells was evaluated by incubating *E. coli* ATCC 25922, a colibactin toxin-producing strain, with the extracts in cell culture for 4 h, using *E. coli* K-12 as a negative control. Tea extracts were tested at concentrations that preserved cell viability above 80%, as determined by the MTT assay. Caco-2 cells were subsequently infected with *E. coli* ATCC 25922 and cocultured with the tea extracts. The results revealed a reduction in the cytopathic effects, such as an increase in cell viability and a decrease in megalocytosis, compared to cells without tea extract treatment. Additionally, all tea extracts increased cell viability while reducing megalocytosis (giant cells) when compared with Caco-2 cells infected with *E. coli* ATCC 25922 at an MOI of 400 (Figure 2 and Table 3). Specifically, the viability of Caco-2 cells significantly increased after cocultivation with *E. coli* ATCC 25922 (MOI 400) when treated with 8 µg/mL oolong tea (90.78 ± 2.76%), 32 µg/mL black tea (87.58 ± 9.74%), and 8 µg/mL or 16 µg/mL green tea (86.15 ± 7.03% and 83.13 ± 1.99%), compared to the viability of Caco-2 cells infected with *E. coli* ATCC 25922 alone (69.74 ± 4.18%). For the phytochemical compounds tested, catechin at concentrations of 1.6 and 3.2 µg/mL significantly protected cell viability against infection, whereas caffeine did not exhibit protective effects. Moreover, *E. coli* K-12 did not affect transient infection in Caco-2 cells (Figure 2 and Table 3).

### 2.5. Inhibition of Colibactin-Induced DNA Damage in Eukaryotic Cells by Treatment with Tea Extracts and Phytochemical Compounds

**Figure 3:**
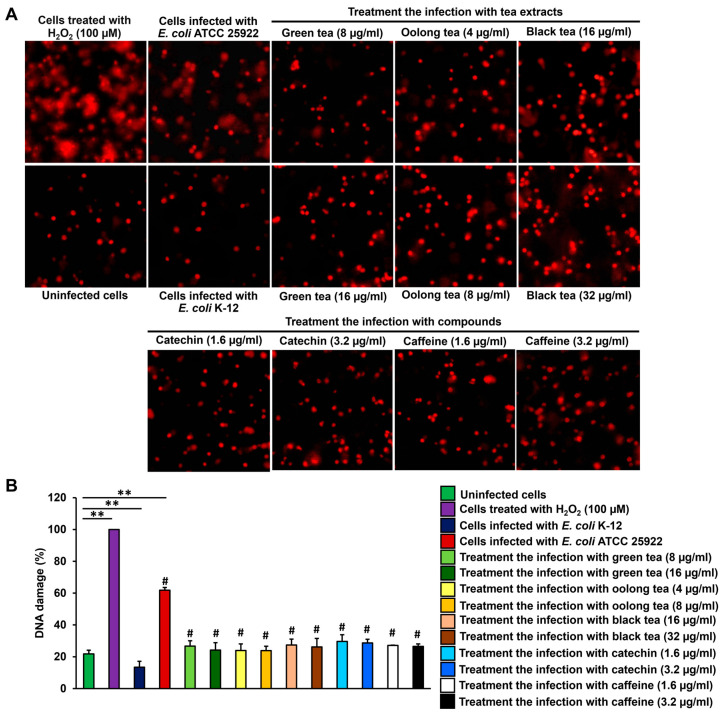
Comet assay (**A**) and percentage of DNA damage (**B**) of Caco-2 cells infected with *E. coli* ATCC 25922 and treatment with tea extracts and compounds. ^#^ Tea extracts and compounds significantly reduce the DNA damage of cells from the infection compared to the *E. coli* ATCC 25922 infection without the treatments (*p* < 0.05). Values are mean ± standard deviation; n = 3 samples. ** indicated *p* < 0.001. All data are used to analyze between two groups using one-way ANOVA and Tukey’s test for multiple comparisons.

The potential of tea extracts to mitigate DNA damage in Caco-2 cells infected with *E. coli* ATCC 25922 was examined. Caco-2 cells were exposed to *E. coli* ATCC 25922 and *E. coli* K-12 at a MOI of 400 for 4 h. DNA damage was detected in the infected cells through the alkaline comet assay, which revealed DNA tails (Figure 3). The degree of DNA damage in cells infected with *E. coli* ATCC 25922 was 61.89 ± 1.67%, compared to 21.83 ± 2.25% in uninfected control cells. Treatment with tea extracts reduced DNA damage in the infected cells, with oolong tea (8 µg/mL) showing significant protective effects, resulting in a DNA damage percentage of 23.84 ± 2.81% (Figure 3). Moreover, green tea (8–16 µg/mL) and black tea (16–32 µg/mL) extracts could inhibit the DNA damage of infected cells at 24.24 ± 4.61–26.72 ± 3.33% and 26.21 ± 5.35–27.36 ± 3.71% (Figure 3). In addition, catechin and caffeine compounds (1.6–3.2 µg/mL) also supported to protect the DNA damage from the infection, with showed the percentage of DNA damage of 26.56 ± 1.50–29.66 ± 4.23% (Figure 3). In this study was used the hydrogen peroxide and *E. coli* K-12 as positive and negative control that represented the DNA damage of 100% and 13.42 ± 3.75%, respectively.

### 2.6. Effect of Colon Cell Cycle After Infection with E. coli-Producing Colibactin Toxin by Treatment with Tea Extracts and Phytochemical Compounds

**Figure 4:**
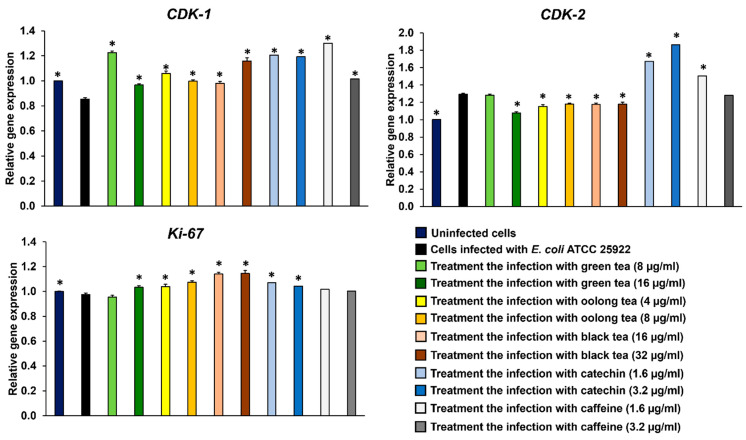
Expression of *CDK-1, CDK-2*, and *Ki-67* genes associated with the cell cycle in Caco-2 cells following infection with colibactin-producing *E. coli* and treatment with tea extracts and compounds. * Data are significantly different compared to cells infected with *E. coli* ATCC 25922 (*p* < 0.05). Values are presented as mean ± standard deviation; n = 3 samples. Statistical analysis between groups was performed using one-way ANOVA and Tukey’s test for multiple comparisons.

After Caco-2 cells were infected with *E. coli* ATCC 25922 for 4 h and treated with tea extracts for 3 days, gene expression related to the cell cycle, including *CDK-1*, *CDK-2*, and *Ki-67*, was detected by qRT-PCR. The results revealed that *E. coli* ATCC 25922 could interfere with the cell cycle arrest of Caco-2 cells by suppressing the gene expression of *CDK-1* and upregulating *CDK-2* compared to uninfected cells (Figure 4). Moreover, all tea extracts could upregulate *CDK-1* gene expression in Caco-2 cells infected with *E. coli* ATCC 25922. Additionally, green tea (16 µg/mL), oolong tea (4 and 8 µg/mL), and black tea (16 and 32 µg/mL) were able to suppress *CDK-2* gene expression compared to Caco-2 cells infected with *E. coli* ATCC 25922 and uninfected cells. These results were correlated with exogenous compounds, catechin and caffeine, which could upregulate the *CDK-1* and *CDK-2* genes (Figure 4). However, the expression of *Ki-67* in Caco-2 cells infected with *E. coli* ATCC 25922 did not differ from that in uninfected cells. The extracts of green tea (16 µg/mL), oolong tea (4 and 8 µg/mL) and black tea (16 and 32 µg/mL), and catechin compound (1.6 and 3.2 µg/mL) were able to upregulate *Ki-67* gene expression in Caco-2 cells during infection (Figure 4).

## 3. Discussion

Most therapeutic drugs used to treat bacterial infections are derived from chemical synthetic agents, which are costly, have greater side effects, and may contribute to the development of antibiotic-resistant bacterial strains [29]. This study identifies promising natural compounds as potential drug candidates via protection from infectious diseases using natural products. Tea has been a popular beverage for centuries, especially green tea, oolong tea, and black tea, which are rich in nutraceuticals, dietary supplements, traditional remedies, pharmaceutical intermediates, and ingredients for synthetic drug development [30]. In this study, extracts from *C. sinensis* leaves, including green tea, oolong tea, and black tea, were prepared using hot water. In the present study, we employed water extraction to investigate the bioactive effects of tea. This approach was chosen because water infusion is the most common and practical method of tea preparation for human consumption, thereby providing results that are more representative of real-life dietary intake. Moreover, water is a non-toxic solvent that ensures the safety and physiological relevance of the extracts [31,32]. Additionally, the extracts were tested against *E. coli* ATCC 25922, a strain known for producing colibactin toxin. This strain carries colibactin genes and is linked to diarrheal diseases and bacteremia [33,34]. The results showed that all tea leaf extracts were effective in inhibiting *E. coli* ATCC 25922, as demonstrated by agar well diffusion. Moreover, tea compounds such as catechin and caffeine have also demonstrated inhibitory effects on *E. coli* that produces colibactin toxin. The antimicrobial activity of tea is due to the presence of catechin polyphenols, which damage the bacterial cell membrane. Epigallocatechin (EGC) and epigallocatechin gallate (EGCG) exhibit the highest antimicrobial effects, with EGCG being the most established in terms of bactericidal activity [35]. In *E. coli*, EGCG induces the destruction of biofilms by damaging bacterial membranes and degrading exopolysaccharides [36]. Oolong tea extract has been shown to exhibit synergistic antibacterial activity against Streptococcus mutans due to its monomeric polyphenols [37]. Additionally, polyphenols in black tea have been found to reduce the expression of virulence traits in clinical isolates of *Shigella dysenteriae* and enteropathogenic *E. coli* (EPEC P2 1265) strains [38]. Green tea has higher levels of catechins compared to oolong, black, and dark teas. Specifically, oolong tea contains about half the amount of EGCG found in green tea, while its polymerized polyphenol content is double that of green tea [39]. Caffeine could inhibit uropathogenic *E. coli* biofilm formation [40]. Tea compounds such as polyphenols and flavonoids can improve gut health by promoting healthy bacterial growth, protecting against bacterial pathogens, and reducing inflammation [41,42]. Moreover, flavonoids such as naringin, naringenin, genistein, kaempferol, anthocyanins, epigallocatechin-3-gallate, and baicalein have been reported to exhibit therapeutic effects for various diseases [43]. However, the other constituents, such as alkaloids, flavonols, and phenolic acids, may also play important roles in tea bioactivity. Comprehensive profiling of tea samples has been reported previously using advanced analytical methods such as UHPLC-DAD, where 20 major compounds were quantified across 121 samples representing six tea types (black tea, green tea, yellow tea, white tea, oolong tea and dark tea). That study demonstrated that the chemical diversity of tea depends strongly on processing and identified biomarkers such as gallocatechin, EGCG, and epicatechin gallate for tea classification [44]. Not only does the type of tea reveal differing contents of catechins and other compounds, but many factors such as species, season, age of the leaves (plucking position), climate, and horticultural conditions (soil, water, mineral fertilizers, etc.) are also relevant [45].

Several studies have reported on the phytochemical compounds in tea leaves and their inhibitory effects on bacterial pathogens. In this study, we evaluated the ability of tea extracts to protect against infection, DNA damage, and cell cycle alterations in colon cells. This research presents a novel report on the effects of tea extracts and the tea compounds catechin and caffeine on *E. coli* ATCC 25922 infections in colon cells. *E. coli* ATCC 25922 produces colibactin, a genotoxin, due to the presence of its *clb* gene cluster responsible for toxin synthesis [34,46]. These toxins can cause DNA double-strand breaks (DSBs) in both in vitro [24] and in vivo studies [25]. To investigate the effects of this toxin, mammalian colon cells (Caco-2) were used and infected with *E. coli* ATCC 25922. The impact on these cells was compared with that of *E. coli* K-12, a strain that does not produce colibactin, at a multiplicity of infection (MOI) of 400 bacteria per cell for 4 h. The morphological changes in the epithelial cells were monitored over a period of 3 days. The results revealed that cytopathic effects on cell morphology, such as megalocytosis (giant cells) and cellular debris, were decreased when treated with tea extracts, catechin and caffeine. Additionally, green tea, oolong tea, black tea, and the tea compounds catechin and caffeine provided significant protection to Caco-2 cells against *E. coli* ATCC 25922 infection, maintaining cell viability above 80% compared to Caco-2 cells infected with *E. coli* ATCC 25922 without the addition of tea extracts or compounds. Zhang et al. [47] showed that oral administration of tea polyphenols (400 mg/kg) alleviated oxidative stress and intestinal damage in C57BL/6 mice with bacterial infection. This protective effect was achieved by inhibiting myeloperoxidase activity and MDA production, as well as by boosting the activity of various intestinal antioxidant enzymes. Catechin-mediated photoprotection of human skin against bacterial infection has also been reported [48]. Moreover, tea catechins, especially EGCG, have been shown to inhibit various viral infections, including HIV, Hepatitis B virus, Rotavirus, and SARS-CoV-2, by interfering with binding protein receptors on host cells [49,50,51,52]. In addition, caffeine can significantly reduce the survival and cytotoxicity of bacteria [40].

Therefore, tea extracts, catechin and caffeine could protect against DNA damage in cells caused by bacterial colibactin (*E. coli* ATCC 25922) infection. These results are supported by both in vitro and in vivo studies. Tea catechins, such as EC, EGC, ECG, and EGCG, can help reduce hydroxyl radical-induced DNA single-strand breaks and base damage by facilitating the rapid chemical repair of DNA radicals. These studies also explain the mechanism through which catechins transfer hydrogen atoms and/or electrons to radical sites on DNA [53,54]. Caffeine can inhibit the DNA damage response (DDR) pathway, which is responsible for repairing DNA breaks and preventing cell division until the damage is resolved [55,56]. Likewise, black tea theaflavins (20 or 50 µM) have been shown to prevent oxidative stress-induced DNA damage in rat normal liver epithelial RL-34 cells [57]. Oral administration of a green tea polyphenol extract equivalent to a human intake of 500 mL of green tea per day for 5 days was found to protect lymphocytes, and to a lesser degree, internal organs like colonocytes and hepatocytes, from oxidative DNA damage in rats [58]. Following infection with bacterial colibactin, *CDK-1* gene expression in Caco-2 cells was found to be downregulated compared to uninfected cells. This reduction in *CDK-1* expression resulted in changes to cell morphology and an increase in the expression of various differentiation markers. Cells with lower *CDK-1* expression exhibited a higher incidence of double-strand breaks (DSBs), failed to activate *CHK2* expression, and could not sustain G2/M cell cycle arrest [59,60]. This phenomenon is associated with elevated DNA damage in Caco-2 cells infected with *E. coli* ATCC 25922. Additionally, the downregulation of *CDK-1* led to the accumulation of cells with abnormal numbers of mitotic organelles, chromosomal abnormalities, and polyploidy [60,61].

Additionally, *CDK-2* gene expression was upregulated in Caco-2 cells infected with *E. coli* ATCC 25922. *CDK-2* is a core cell-cycle regulator that is active from the late G1 phase throughout the S phase [62]. In addition to promoting cell cycle progression, *CDK-2* has been described as playing a positive role in cell cycle arrest during the DNA damage response, particularly at the G2/M checkpoint [63]. Accumulating evidence indicates that CDK-2 promotes hyperproliferation of cells and induces the progression of cancer cells [64]. This study confirms previous research showing that colibactin toxin induces DNA double-strand breaks, chromosome aberrations, and cell cycle arrest at the G2/M phase [24,25]. Moreover, infection with *E. coli*-producing colibactin toxin has been shown to increase the number of tumors in various colorectal cancer (CRC) mouse models [65,66]. This study highlights new findings that tea extracts, including green tea, oolong tea, and black tea, as well as the tea compounds catechin and caffeine, can maintain the normal cell cycle of colon cells infected with *E. coli*-producing colibactin toxin through the modulation of *CDK-1* and *CDK-2*. Furthermore, tea extracts and compounds, particularly catechin, were found to promote the proliferation of colon cells infected with *E. coli*-producing colibactin toxin, as evidenced by the upregulation of *Ki-67* gene expression. The expression of *Ki-67* is strongly associated with cell proliferation and growth [67]. Overall, the findings suggest that tea extracts and tea compounds exhibit potent antimicrobial and cytoprotective properties, making them promising candidates for preventing or mitigating colibactin-associated infections. Further studies are warranted to elucidate the mechanisms underlying these effects and to explore the clinical applications of tea extracts in combating bacterial infections and related diseases. For safety reasons, consuming tea catechins and caffeine from a typical cup of tea is generally considered safe for most people. However, the safe amount of catechins and caffeine varies among individuals. Exceeding the recommended daily intake of approximately 400 mg is not advised [68].

## 4. Materials and Methods

### 4.1. Tea Extraction

Dried tea leaves, including green tea (batch number: 170323), oolong tea (batch number: 200323), and black tea (batch number: 211122), were sourced from the Royal Project Foundation, Chiang Mai, Thailand, a government-supported project that complies with national agricultural regulations. No wild plant collection was performed for this study. The leaves were extracted using hot distilled water (100 °C) at a 1:10 (*w*/*v*) ratio for 1 h, with the process repeated three times [69]. The resulting extract was filtered through Whatman No. 1 filter paper, and the filtrate was concentrated using a rotary evaporator (BÜCHI, Flawil, Switzerland) at 50 °C under a reduced pressure of 50 mbar. It was then dried via freeze drying (LABCONCO, Kansas, MO, USA). The dried extracts were stored at −20 °C and dissolved in sterile deionized water at a concentration of 500 mg/mL prior to use. Tea extracts at this high concentration (500 mg/mL) were used in the antibacterial assay to evaluate maximal bacterial inhibition, whereas lower concentrations were used in cell infection experiments to avoid cytotoxicity.

### 4.2. High Performance Liquid Chromatography (HPLC)

High-performance liquid chromatography (HPLC) was employed to determine the levels of catechin, EGCG, caffeine, and theaflavin in tea extracts. All standard compounds were purchased from Sigma-Aldrich (Darmstadt, Germany). The extracts and standard compounds were filtered through a 0.45 µm sterile microfilter. Subsequently, 20 µL of the filtered sample was injected into the HPLC system (Agilent Technologies 1200 series, Santa Clara, CA, USA). Analysis was conducted using a UV detector set at 276 nm with an Agilent Eclipse XDB-C18 column (4.6 × 150 mm, 5 µm). The mobile phase used for separation consisted of mobile phase A (deionized water) and mobile phase B (methanol, RCl Labscan, Bangkok, Thailand). Gradient elution was performed with varying ratios of mobile phases A and B as follows: 100:0 at 0 min, 50:50 at 15 min, and 0:100 at 30 min. The HPLC conditions included a flow rate of 1.0 mL/min and a total run time of 30 min. The concentrations of catechin, EGCG, caffeine, and theaflavin in the tea extracts were quantified by comparison with the corresponding standard compounds.

### 4.3. Bacterial Strains

The standard strain of *E. coli* ATCC 25922 and *E. coli* K-12 strain BW25113 were procured from Department of Biotechnology, College of Life Sciences, Ritsumeikan University, Kusatsu, Shiga, Japan.

### 4.4. Agar Well Diffusion Assay

The bacterial inhibition assay was performed using the agar well diffusion method [70]. Bacterial strains were cultured in Mueller–Hinton (MH) broth (HiMedia, Maharashtra, India) and incubated at 37 °C for 18–24 h. The bacterial suspensions were adjusted to a McFarland standard No. 0.5 (10^8^ CFU/mL, OD_600_ = 0.08–0.1) and spread evenly onto Mueller Hinton agar (MHA). Wells were created using a 10 mm cork borer, and 100 µL of sample was placed in each well. After an incubation period, the inhibition zone diameter was measured. Gentamycin (Bio Basic Inc., Amherst, NY, USA) at a concentration of 1 mg/mL was used as a positive control.

### 4.5. Cytotoxicity Assay

The cytotoxicity of tea extracts or compounds was evaluated using the MTT assay. Human colon (Caco-2) cells were purchased from the Cell Engineering Division, RIKEN BioResource Research Center (BRC) (Cell No. RCB0988, Ibaraki, Japan) and cultured in Dulbecco’s Modified Eagle Medium (DMEM; Gibco, Grand Island, NY, USA) supplemented with 10% (*v*/*v*) heat-inactivated fetal bovine serum (HyClone™, Pittsburgh, PA, USA), 100 Units/mL penicillin, and 100 µg/mL streptomycin (CAISSON, Smithfield, UT, USA). The cells were incubated at 37 °C in a 5% CO_2_ incubator (SHEL LAB, Cornelius, OR, USA), then washed twice with phosphate-buffered saline (PBS, pH 7.4) and trypsinized using 0.05% (*v*/*v*) trypsin-EDTA solution (CAISSON, Smithfield, UT, USA). Caco-2 cells (5 × 10^4^ cells/mL) were seeded into 96-well plates and incubated at 37 °C in a 5% CO_2_ incubator for 24 h. After incubation, different concentrations of the samples were added to the wells, and the plates were further incubated for 48 h under the same conditions. MTT solution (Bio Basic Inc., Amherst, NY, USA) was then added to each well and incubated for 4 h. The resulting blue formazan crystals were dissolved in dimethyl sulfoxide (DMSO), and absorbance was measured at 540 nm and 630 nm using a microplate reader (EZ Read 2000, Biochrom, Cambridge, UK). Cell viability was calculated as a percentage by comparing the absorbance values of treated cells to those of the control group [71].

### 4.6. Transient Infection and Treatment

Caco-2 cells (5 × 10^4^ cells/mL) were seeded in a 96-well plate (Corning, New York, NY, USA) and incubated for 24 h. *E. coli* ATCC 25922 (colibactin-producing) and *E. coli* K-12 (non-colibactin-producing) were grown in Tryptic Soy Broth (TSB) (HiMedia, Maharashtra, India) for 8–12 h. The bacteria were harvested by centrifuge (Hettich GmbH & Co. KG, Tuttlingen, Germany) at 6000 rpm for 5 min, washed twice with PBS, and resuspended in DMEM at a concentration of 5 × 10^8^ CFU/mL (OD_600_ = 0.4–0.5). Caco-2 cells were washed with PBS and infected at a multiplicity of infection (MOI) of 400 with the bacterial strains in the presence of tea extracts or compounds. Sample concentrations were selected to maintain cell viability above 80%. The plate was incubated at 37 °C in a 5% CO_2_ environment for 4 h. After infection, cells were washed with PBS to remove bacteria and further incubated with DMEM supplemented with 10% fetal bovine serum and 200 µg/mL gentamicin for 3–4 days.

Cells were washed with PBS, fixed with 4% formaldehyde (Sigma-Aldrich, Darmstadt, Germany) in PBS for 20 min, and stained with 1% methylene blue (TPC™, Thailand Pharmaceutical Chemical Co., Ltd., Bangkok, Thailand) for 1 h. Morphological observations were conducted using an inverted fluorescence microscope (ECLIPSE Ts2R-FL, Nikon, Tokyo, Japan). Uninfected cells without treatment displayed a confluent monolayer, whereas cells infected with *E. coli* ATCC 25922 showed a cytopathic phenotype characterized by reduced cell density, megalocytosis, and markers of cell death such as apoptotic bodies and cell debris, as reported by Bossuet-Greif et al. [72]. Cell viability was quantified using the MTT assay following the protocol of Umthong et al. [71].

### 4.7. DNA Damage by Alkaline Comet Assay

The inhibition of DNA damage in Caco-2 colon cells was evaluated following infection with the colibactin-producing strain *E. coli* ATCC 25922 and subsequent treatment with tea extracts or compounds. This was analyzed using the alkaline comet assay as described by Kothandapani et al. [73] and Sawant et al. [74]. Caco-2 cells (5 × 10^4^ cells/mL) were cultured in DMEM at 37 °C in 5% CO_2_ for 24 h. Samples at non-toxic concentrations (maintaining over 80% cell viability) were introduced to the cultures, followed by infection with *E. coli* at a multiplicity of infection (MOI) of 400. Hydrogen peroxide (100 µM) served as a positive control for DNA damage. Plates were incubated for 4 h, then cells were washed, trypsinized, and collected by centrifugation at 3000 rpm and 4 °C for 5 min.

Cells were resuspended in PBS, counted, and adjusted to 10,000 cells. Each sample was mixed with 1% low melting point agarose, layered onto slides pre-coated with 1% agarose, and topped with 0.5% low melting point agarose. After solidification, slides were lysed in buffer; 2.5 M NaCl (RCl Labscan, Bangkok, Thailand), 10 mM Tris (Research Organics, Cleveland, OH, USA), 100 mM EDTA (VWR, Leuven, Belgium), 1% Triton X-100 (Amresco, Solon, OH, USA), pH 10 for 1 h at 4 °C in the dark, then placed in an electrophoresis tank with alkaline buffer; 50 mM NaOH (RCl Labscan, Bangkok, Thailand), 1 mM EDTA (VWR, Belgium), pH > 12) at 4 °C. Electrophoresis (Cleaver Scientific Ltd., Rugby, Warwickshire, UK) was performed at 0.7 V/cm and 300 mA for 30 min. Afterward, slides were neutralized in 0.4 M Tris-HCl buffer (pH 7.5) for 10 min, stained with propidium iodide (PI, Abcam, Cambridge, MA, Middlesex) for 30 min in the dark, and observed using an inverted fluorescence microscope. DNA comet tails from a minimum of 200 cells per slide were analyzed, and the percentages of DNA damage were calculated and compared to the uninfected control cells.

### 4.8. Quantitative Real-Time Polymerase Chain Reaction (qRT-PCR)

The mRNA expression of cell cycle-related genes, including *CDK-1*, *CDK-2*, and *Ki-67,* was analyzed following infection and treatment with tea extracts or compounds using qRT-PCR. Caco-2 cells (2 × 10^5^ cells/mL) were cultured in DMEM at 37 °C in a 5% CO_2_ incubator for 24 h. Tea extracts were introduced to the cell culture, then infected with *E. coli* ATCC 25922 (a colibactin-producing strain) at a multiplicity of infection (MOI) of 400 for a duration of 4 h. Bacterial growth was then inhibited using gentamicin, and the plates were incubated for 3 days at 37 °C in a 5% CO_2_ environment. After incubation, cells were harvested and washed three times with 1X PBS prior to RNA extraction. Total mRNA from the Caco-2 cells was extracted using TRIzol™ reagent (Invitrogen, Carlsbad, CA, USA) and converted to cDNA using the ReverTra Ace^®^ qPCR RT Master Mix (TOYOBO, Osaka, Japan). The qPCR reaction mixture included 100 ng of cDNA, 2X SensiFAST SYBR^®^ No-ROX Mix (BIOLINE, London, UK), and primers at a concentration of 400 nM each (forward and reverse). The realtime PCR was conducted in QIAquant real-time PCR cyclers (Qiagen, Venlo, Netherlands). The qPCR conditions were as follows: initial denaturation at 95 °C for 2 min, followed by 40 cycles of denaturation at 95 °C for 5 s and annealing/extension at 60 °C for 30 s. Cycle threshold (Ct) values for the target genes were normalized to the internal control gene GAPDH to determine relative expression levels. Primer sequences for the target genes were designed based on previously published studies [75,76,77]. *CDK-1* (forward primer: 5′-GTCCGCAACAGGGAAGAACAG-3′, reverse primer: 5′-CGAAAGCCAAGATAAGCAACTCC-3′), *CDK-2* (forward primer: 5′-TATGAGTCAAAAGGCGGGGT-3′, reverse primer: 5′-TGCACTCATCCACCAATCCT-3′) and *Ki-67* (forward primer: 5′-GTTGGTCTCGCGTAAGTCAA-3′, reverse primer: 5′-CAGACTCCACGTCTCTTCCC-3′).

### 4.9. Statistical Analysis

All experiments were conducted independently on three separate occasions. Data were expressed as mean ± SD values of the independent samples. Statistical analysis of the results from both treatment and control groups was performed using T-tests and ANOVA in IBM SPSS Statistics 20.

## 5. Conclusions

**Figure 5:**
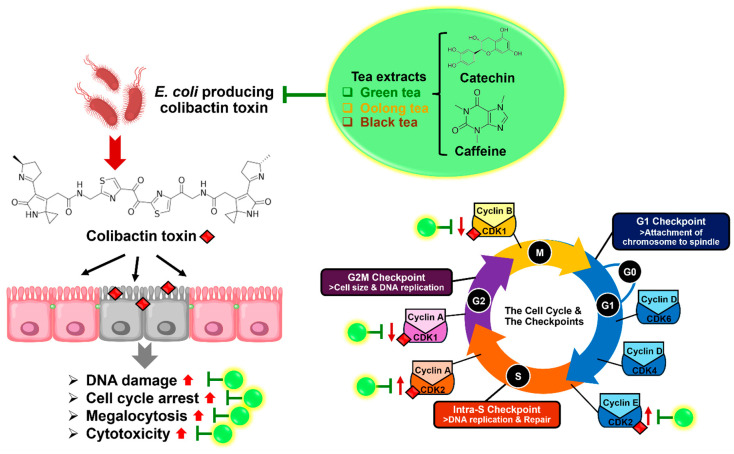
Proposed mechanism of action by which tea extracts (green, oolong, and black tea) and tea compounds (catechin and caffeine) inhibit *E. coli* colibactin toxin infection in colon cells. Tea extracts and compounds showed strong antimicrobial activity against colibactin-producing *E. coli*. They protected Caco-2 cells from cytopathic effects (megalocytosis, cell debris), improved cell viability, and reduced colibactin-induced DNA damage. *E. coli* infection downregulated *CDK-1* and upregulated *CDK-2*, causing cell cycle arrest and DNA damage. Tea extracts and compounds restored normal cell cycle regulation (↑*CDK-1*, ↓*CDK-2*) and catechin promoted cell proliferation via *Ki-67* expression.

This study highlights the potential of tea extracts, particularly green tea, oolong tea and black tea, and tea compounds (catechin and caffeine), as natural agents for combating bacterial infections, specifically those caused by *E. coli* ATCC 25922, a colibactin-producing strain. These tea extracts and tea compounds demonstrated significant antimicrobial activity against *E. coli*-producing colibactin toxins. Furthermore, tea extracts and catechin protected colon cells (Caco-2) from cytopathic effects, including megalocytosis and cell debris, induced by *E. coli* ATCC 25922 infection. All tea extracts and tea compounds also promoted higher cell viability and reduced DNA damage caused by colibactin-induced double-strand breaks. In terms of the cell cycle, the study revealed that *E. coli* ATCC 25922 downregulated *CDK-1* and upregulated *CDK-2* in Caco-2 cells, leading to cell cycle arrest and increased DNA damage. Interestingly, treatment with tea extracts and tea compounds restored normal cell cycle function by upregulating *CDK-1* and suppressing *CDK-2* expression, while tea extracts and catechin promoted cell proliferation via *Ki-67* expression (Figure 5). Overall, this research demonstrates the therapeutic potential of tea extracts as a natural alternative to synthetic antimicrobial agents. They not only inhibit bacterial infection but also protect host cells from the detrimental effects of *E. coli*-producing colibactin toxin, making them promising candidates for future treatment strategies against bacterial infections and related diseases.

## Figures and Tables

**Table 1:** Phytochemical compounds contents in tea extracts.

Tea Extracts	Chemical Compound Contents (mg/mL)			
	Catechin	EGCG	Caffeine	Theaflavin
Green tea	12.680 ± 0.019 ^a^	37.622 ± 0.163 ^a^	56.909 ± 0.444 ^a^	ND ^a^
Oolong tea	233.475 ± 13.320 ^b^	251.839 ± 8.751 ^b^	2.658 ± 0.053 ^b^	2.192 ± 0.297 ^b^
Black tea	ND ^c^	269.714 ± 7.860 ^c^	ND ^c^	ND ^c^

The data represented with different superscript letters (a, b, c), are expressed as mean ± SD from three independent experiments and indicate significant differences in the chemical compound contents among the tea extracts (*p* < 0.05). ND: not detected.

**Table 2:** Antibacterial activity by agar well diffusion.

Samples	Concentration (mg/mL)	Zone of Inhibition (mm) *	
		*E. coli* ATCC 25922	*E. coli* K-12
Tea extracts			
Green tea	500	16.3 ± 4.5	15.0 ± 1.7
Oolong tea	500	18.3 ± 0.5	16.0 ± 1.0
Black tea	500	13.7 ± 1.3	14.0 ± 1.0
Phytochemical compounds			
Catechin	5.0	16.3 ± 0.6	15.7 ± 0.6
Caffeine	5.0	19.3 ± 0.6	19.0 ± 0.0
Gentamycin	1.0	23.8 ± 0.5	26.3 ± 0.6

* The results are presented as mean ± SD of triplicate independent experiments.

**Table 3:** Viability of cells after treatment with tea extracts during infection of Caco-2 cells with *E. coli* ATCC 25922 and *E. coli* K-12 at MOI of 400 for 4 h.

Samples	*E. coli* ATCC 25922 Infection with Caco-2 Cells		*E. coli* K-12 Infection with Caco-2 Cells	
	Concentration (µg/mL)	Viability of Cells (%) *	Concentration (µg/mL)	Viability of Cells (%) *
Tea extracts				
Green tea	8	86.15 ± 7.03 ^b^	8	80.58 ± 16.05 ^a^
	16	83.13 ± 1.99 ^b^	16	86.47 ± 12.59 ^a^
Oolong tea	4	87.93 ± 14.22 ^a^	4	88.01 ± 8.03 ^a^
	8	90.78 ± 2.76 ^b^	8	82.31 ± 11.49 ^a^
Black tea	16	81.42 ± 9.96 ^a^	16	86.98 ± 11.88 ^a^
	32	87.58 ± 9.74 ^b^	32	88.28 ± 11.25 ^a^
Phytochemical compounds				
Catechin	1.6	81.77 ± 9.47 ^b^	1.6	80.97 ± 7.04 ^a^
	3.2	85.59 ± 9.74 ^b^	3.2	84.49 ± 7.13 ^a^
Caffeine	1.6	76.43 ± 13.64 ^a^	1.6	83.58 ± 11.42 ^a^
	3.2	79.81 ± 6.46 ^a^	3.2	85.29 ± 9.19 ^a^
No treatment	-	69.74 ± 4.18 ^a^	-	78.92 ± 0.62 ^a^

* The data represented with different superscript letters (a, b) are expressed as mean ± SD from three independent experiments and indicate significant differences in cell viability percentages after treatment with tea extracts or compounds compared to untreated controls (*p* < 0.05). Statistical analysis between groups was performed using one-way ANOVA, followed by Tukey’s test for multiple comparisons.

## Data Availability

The datasets used and analyzed are available from the corresponding author upon reasonable request.

## Supplementary Materials

The following supporting information can be downloaded at: https://www.mdpi.com/article/10.3390/antibiotics14090886/s1, Figure S1: Antibacterial activity of tea extracts; green tea (G), oolong tea (O), black tea (B), and compounds; catechin (CC) and caffeine (CF) against *E. coli* K-12 (**A**,**C**) and *E. coli* ATCC 25922 (**B**,**D**) by agar well diffusion assay. Sterile distilled water (N) and gentamycin (P) were used as a negative and positive controls for bacterial inhibition.
