# Supplementary material for: Antimicrobial and Cytoprotective Effects of Tea Extracts Against Escherichia coli-Producing Colibactin Toxin Infections
Source: Antibiotics (Basel). 2025 Sep 2;14(9):886. doi: 10.3390/antibiotics14090886 (PMC12466732; doi:10.3390/antibiotics14090886)
Supplement: Supplementary file 1 [file antibiotics-14-00886-s001.zip › antibiotics-3826560-supplementary.pdf]

Supplementary Materials

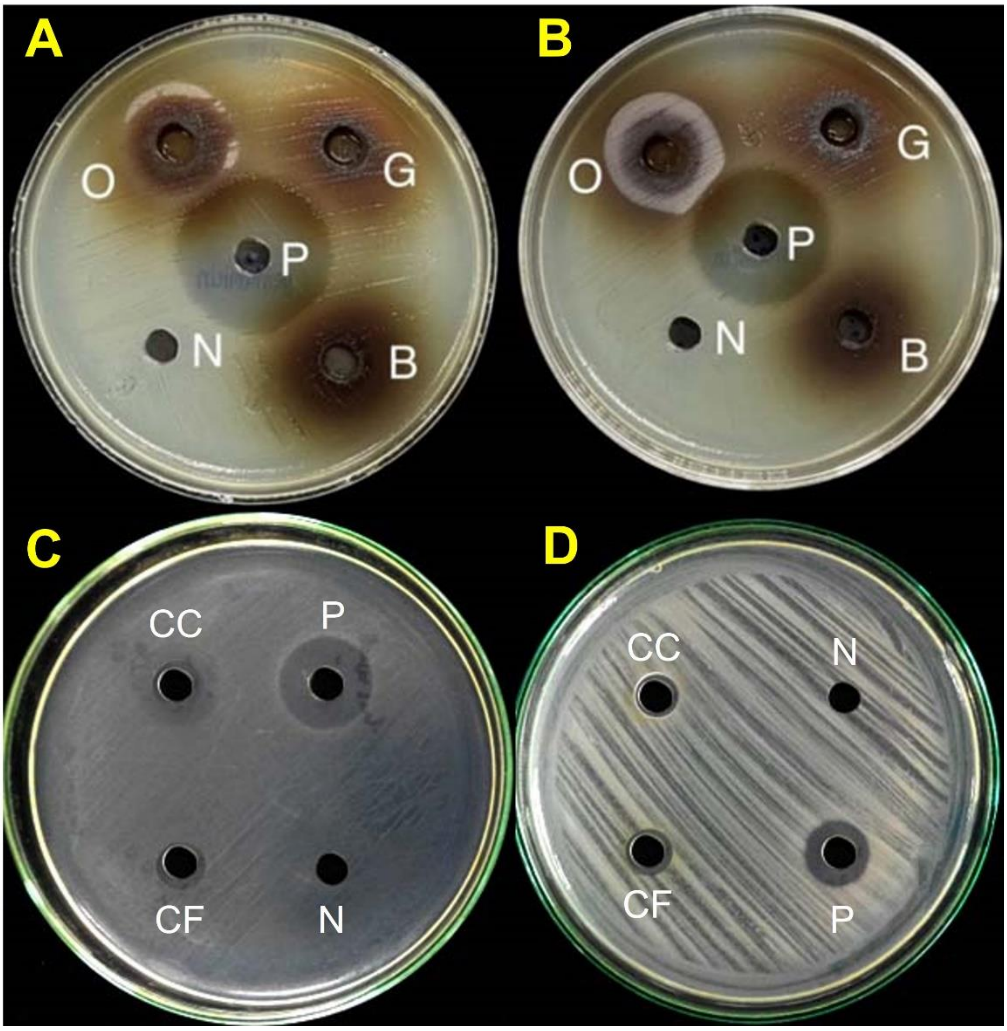

Figure S1. Antibacterial activity of tea extracts; green tea (**G**), oolong tea (**O**), black tea (**B**), and compounds; catechin (**CC**) and caffeine (**CF**) against *E. coli* K-12 (**A** and **C**) and *E. coli* ATCC 25922 (**B** and **D**) by agar well diffusion assay. Sterile distilled water (**N**) and gentamycin (**P**) were used as a negative and positive controls for bacterial inhibition.
